# Author Correction: Spin-controlled generation of indistinguishable and distinguishable photons from silicon vacancy centres in silicon carbide

**DOI:** 10.1038/s41467-021-25869-w

**Published:** 2021-10-07

**Authors:** Naoya Morioka, Charles Babin, Roland Nagy, Izel Gediz, Erik Hesselmeier, Di Liu, Matthew Joliffe, Matthias Niethammer, Durga Dasari, Vadim Vorobyov, Roman Kolesov, Rainer Stöhr, Jawad Ul-Hassan, Nguyen Tien Son, Takeshi Ohshima, Péter Udvarhelyi, Gergő Thiering, Adam Gali, Jörg Wrachtrup, Florian Kaiser

**Affiliations:** 1 3rd Institute of Physics, University of Stuttgart and Institute for Quantum Science and Technology (IQST), 70569 Stuttgart, Germany; 2 Advanced Research and Innovation Center, DENSO CORPORATION, Nisshin, 470-0111 Japan; 3 Department of Physics, Chemistry and Biology, Linköping University, SE-58183 Linköping, Sweden; 4 National Institutes for Quantum and Radiological Science and Technology, Takasaki, 370-1292 Japan; 5 Department of Biological Physics, Eötvös University, Pázmány Péter sétány 1/A, Budapest, 1117 Hungary; 6 Wigner Research Centre for Physics, P.O. Box 49, Budapest, 1525 Hungary; 7 Department of Atomic Physics, Budapest University of Technology and Economics, Budafoki út 8., Budapest, 1111 Hungary

**Keywords:** Quantum optics, Single photons and quantum effects, Semiconductors, Qubits

Correction to: *Nature Communications* 10.1038/s41467-020-16330-5, published online 20 May 2020.

The original version of this Article contained an error in Supplementary Eq. (8), and incorrectly read:

$$V=\frac{1}{{(1-\varepsilon )}^{2}}\left[\left\{{\left(\frac{{{{{{{{\rm{SN}}}}}}}}+1}{{{{{{{{\rm{SN}}}}}}}}}\right)}^{2}{\alpha }_{2}+\frac{2{\alpha }_{1}}{{{{{{{{\rm{SN}}}}}}}}}\right\}-(1-{V}_{0})\left\{{\left(\frac{{{{{{{{\rm{SN}}}}}}}}+1}{{{{{{{{\rm{SN}}}}}}}}}\right)}^{2}{\alpha }_{1}+\frac{2{\alpha }_{2}}{{{{{{{{\rm{SN}}}}}}}}}\right\}\right]$$

The correct form of Supplementary Eq. (8) is:

$$V=\frac{1}{{(1-\varepsilon )}^{2}}\left[\left\{{\left(\frac{{{{{{{{\rm{SN}}}}}}}}+1}{{{{{{{{\rm{SN}}}}}}}}}\right)}^{2}{\alpha }_{2}+\frac{4{\alpha }_{1}}{{{{{{{{\rm{SN}}}}}}}}}\right\}-(1-{V}_{0})\left\{{\left(\frac{{{{{{{{\rm{SN}}}}}}}}+1}{{{{{{{{\rm{SN}}}}}}}}}\right)}^{2}{\alpha }_{1}+\frac{2{\alpha }_{2}}{{{{{{{{\rm{SN}}}}}}}}}\right\}\right]$$

The original version of this Article contained an error in Supplementary Eq. (11), and incorrectly read:

$$V=\frac{1}{{(1-\varepsilon )}^{2}{\beta }_{{{{{{{{\rm{jitter}}}}}}}}}}\left[\left\{{\left(\frac{{{{{{{{\rm{SN}}}}}}}}+1}{{{{{{{{\rm{SN}}}}}}}}}\right)}^{2}{\alpha }_{2}+\frac{2{\alpha }_{1}}{{{{{{{{\rm{SN}}}}}}}}}\right\}-(1-{V}_{0})\left\{{\left(\frac{{{{{{{{\rm{SN}}}}}}}}+1}{{{{{{{{\rm{SN}}}}}}}}}\right)}^{2}{\alpha }_{1}+\frac{2{\alpha }_{2}}{{{{{{{{\rm{SN}}}}}}}}}\right\}\right]$$

The correct form of Supplementary Eq. (11) is:

$$V=\frac{1}{{(1-\varepsilon )}^{2}{\beta }_{{{{{{{{\rm{jitter}}}}}}}}}}\left[\left\{{\left(\frac{{{{{{{{\rm{SN}}}}}}}}+1}{{{{{{{{\rm{SN}}}}}}}}}\right)}^{2}{\alpha }_{2}+\frac{4{\alpha }_{1}}{{{{{{{{\rm{SN}}}}}}}}}\right\}-(1-{V}_{0})\left\{{\left(\frac{{{{{{{{\rm{SN}}}}}}}}+1}{{{{{{{{\rm{SN}}}}}}}}}\right)}^{2}{\alpha }_{1}+\frac{2{\alpha }_{2}}{{{{{{{{\rm{SN}}}}}}}}}\right\}\right]$$

As a result of the errors identified in Supplementary Eqs. (8) and (11), the original version of this Article contained an error in Figs. 3 and 4, and Supplementary Fig. 4.

The correct version of Fig. 3 is:



Which replaces the previous incorrect version.

The correct version of Fig. 4 is:



Which replaces the previous incorrect version.

The correct version of Supplementary Fig. 4 is: 

which replaces the previous incorrect version.

As a result of the errors identified in Supplementary Eqs. (8) and (11), the original version of the Supplementary Information contained an error in Supplementary Table 1. The correct version of Supplementary Table 1 is:

**Table:** 

		Pure dephasing limited		Spectral diffusion limited	
Temperature	PLE linewidth	${\gamma }_{{{{{{{{\rm{max }}}}}}}}}^{{\prime} }/2{{\pi }}$	${\Gamma }_{0}^{{\prime} }/2{{\pi }}$	${\Gamma }_{0,\,{{{{{{{\rm{max }}}}}}}}}^{{\prime} }/2{{\pi }}$	${\tau }_{{{{{{{{\rm{c}}}}}}}},{{{{{{{\rm{min }}}}}}}}}$
[K]	[MHz]	[MHz]	[MHz]	[MHz]	[ns]
5.0	$62.4\pm 0.4$	$1.5\pm 0.4$	$34.4\pm 0.5$	$35.9\pm 0.4$	$109\pm 8$
5.9	$70.1\pm 0.3$	$4.0\pm 0.7$	$39.6\pm 0.7$	$43.6\pm 0.3$	$81\pm 6$
6.8	$82.4\pm 0.3$	$11.5\pm 1.7$	$44.4\pm 1.8$	$55.9\pm 0.3$	$51\pm 6$

,which replaces the previous incorrect version.

As a result of the errors identified in Supplementary Eqs. (8) and (11), the original version of the Article contained the errors listed in the second column of the table below.

The corrected values (listed in the third column) have been corrected both in the PDF and HTML versions of the Article.

**Table:** 

Position in the Article	Wrong items	Corrected items
Introduction	… a naturally stable spin-photonic interface at temperatures up to 6.6 K	… a naturally stable spin-photonic interface at temperatures up to 6.9 K
Results, Spin-controlled distinguishable photon generation	${V}_{{{{{{{{\rm{max }}}}}}}}}=0.65\pm 0.05\,$	${V}_{{{{{{{{\rm{max }}}}}}}}}=0.73\pm 0.05$
	${V}_{{{{{{{{\rm{norm}}}}}}}},\pi /2}=\frac{{V}_{{{{{{{{\rm{measured}}}}}}}}}}{{V}_{{{{{{{{\rm{max }}}}}}}}}}=0.61\pm 0.10$	${V}_{{{{{{{{\rm{norm}}}}}}}},\pi /2}=\frac{{V}_{{{{{{{{\rm{measured}}}}}}}}}}{{V}_{{{{{{{{\rm{max }}}}}}}}}}=0.65\pm 0.07$
	${V}_{{{{{{{{\rm{norm}}}}}}}},\pi /4}=0.89\pm 0.10$	${V}_{{{{{{{{\rm{norm}}}}}}}},\pi /4}=0.90\pm 0.09$
	${V}_{{{{{{{{\rm{norm}}}}}}}},3\pi /4}=0.37\pm 0.26$	${V}_{{{{{{{{\rm{norm}}}}}}}},3\pi /4}=0.47\pm 0.17$
Results, Temperature stability of spin-photonic properties	$A=2\pi \cdot (365\pm 36)\,{{{{{{{\rm{MHz}}}}}}}}{({{{{{{{\rm{meV}}}}}}}})}^{-3}$	$A=2\pi \cdot (237\pm 12)\,{{{{{{{\rm{MHz}}}}}}}}{({{{{{{{\rm{meV}}}}}}}})}^{-3}$
	${T}_{{{{{{{{\rm{crit}}}}}}}}} > 6.6$ K	${T}_{{{{{{{{\rm{crit}}}}}}}}} >$6.9 K
Figure 3c, caption	… the fringe pattern with the expected modulation at $0.966\pm 0.007$ GHz	… the fringe pattern with the expected modulation at $0.965\pm 0.006$ GHz
Figure 4b, caption	… the theoretical expectation$\,(0.86\pm 0.01)$	… the theoretical expectation$\,(0.80\pm 0.01)$

As a result of the errors identified in Supplementary Eqs. (8) and (11), the original version of the Supplementary Information contained the errors listed in the second column of the table below.

The HTML has been updated to include a corrected version of the Supplementary Information.

**Table:** 

Position in Supplementary Information	Wrong items	Corrected items
Supplementary Note 3, page 7	… the maximum achievable HOM visibility is upper bound at 87%	… the maximum achievable HOM visibility is upper bound at 81%
Supplementary Note 3, page 8	… the maximum achievable HOM visibility is upper bound at $(86\pm 1)$%	… the maximum achievable HOM visibility is upper bound at $(80\pm 1)$%
Supplementary Note 4, page 11, Supplementary Fig. 6, caption	$V({{{{{{{\rm{RF}}}}}}}}\,{{{{{{{\rm{before}}}}}}}}\,{{{{{{{\rm{sequence}}}}}}}})=0.65\pm 0.05$	$V({{{{{{{\rm{RF}}}}}}}}\,{{{{{{{\rm{before}}}}}}}}\,{{{{{{{\rm{sequence}}}}}}}})=0.73\pm 0.05$
Supplementary Note 7, page 17	$\gamma =(119\pm 5)\,{{{{{{{\rm{MHz}}}}}}}}$	$\gamma =(84\pm 4)\,{{{{{{{\rm{MHz}}}}}}}}$
	$(1-{V}_{{{{{{{{\rm{norm}}}}}}}}})=0.39\pm 0.1$	$(1-{V}_{{{{{{{{\rm{norm}}}}}}}}})=0.37\pm 0.06$
	$\frac{{c}_{2}}{{c}_{1}}=\frac{{V}_{{{{{{{{\rm{norm}}}}}}}}}}{1-{V}_{{{{{{{{\rm{norm}}}}}}}}}}=1.55\pm 0.47$	$\frac{{c}_{2}}{{c}_{1}}=\frac{{V}_{{{{{{{{\rm{norm}}}}}}}}}}{1-{V}_{{{{{{{{\rm{norm}}}}}}}}}}=1.68\pm 0.45$
	${c}_{3}/{c}_{1}=0.60\pm 0.12$	${c}_{3}/{c}_{1}=0.63\pm 0.11$
	${c}_{1}=739\pm 11$	${c}_{1}=753\pm 12$
	$\delta \nu =(0.966\pm 0.007)\,{{{{{{{\rm{GHz}}}}}}}}$	$\delta \nu =(0.965\pm 0.006)\,{{{{{{{\rm{GHz}}}}}}}}$
	${\sigma }_{{{{{{{{\rm{det }}}}}}}}}=(0.16\pm 0.02)\,{{{{{{{\rm{ns}}}}}}}}$	${\sigma }_{{{{{{{{\rm{det }}}}}}}}}=(0.17\pm 0.02)\,{{{{{{{\rm{ns}}}}}}}}$

The Supplementary Information contained a typographical error in the second sentence of the caption of Supplementary Fig. 6, and incorrectly read:

“Time gating settings are ${t}_{{{{{{{{\rm{Start}}}}}}}}}=2\,{{{{{{{\rm{ns}}}}}}}}$ and Δ*t* = 16.5 ns”.

The correct sentence is:

“Time gating settings are ${t}_{{{{{{{{\rm{Start}}}}}}}}}=2\,{{{{{{{\rm{ns}}}}}}}}$ and Δ*t* = 16 ns”.

The HTML has been updated to include a corrected version of the Supplementary Information. The correct version of the Supplementary Information can be found associated with this Correction.

## Supplementary information

Supplementary Information

