# Supplementary material for: Spin-controlled generation of indistinguishable and distinguishable photons from silicon vacancy centres in silicon carbide
Source: Nat Commun. 2020 May 20;11:2516. doi: 10.1038/s41467-020-16330-5 (PMC7239935; doi:10.1038/s41467-020-16330-5)
Supplement: Supplementary file 1 — Supplementary Information [file 41467_2020_16330_MOESM1_ESM.pdf]

Supplementary Information

Spin-controlled generation of indistinguishable and distinguishable photons from silicon vacancy centres in silicon carbide

Morioka et al.

Supplementary Information: Spin-controlled generation of indistinguishable and distinguishable photons from silicon vacancy centres in silicon carbide

Naoya Morioka[1,2,*], Charles Babin[1], Roland Nagy[1], Izel Gediz[1], Erik Hesselmeier[1], Di Liu[1], Matthew Joliffe[1], Matthias Niethammer[1], Durga Dasari[1], Vadim Vorobyov[1], Roman Kolesov[1], Rainer Stöhr[1], Jawad Ul-Hassan[3], Nguyen Tien Son[3], Takeshi Ohshima[4], Péter Udvarhelyi[5,6,7], Gergő Thiering[6], Adam Gali[6,7], Jörg Wrachtrup[1], and Florian Kaiser[1,#]

* n.morioka@pi3.uni-stuttgart.de

# f.kaiser@pi3.uni-stuttgart.de

1. 3rd Institute of Physics, University of Stuttgart and Institute for Quantum Science and Technology IQST, 70569, Stuttgart, Germany
2. Advanced Research and Innovation Center, DENSO CORPORATION, 470-0111, Nisshin, Japan
3. Department of Physics, Chemistry and Biology, Linköping University, SE-58183, Linköping, Sweden
4. National Institutes for Quantum and Radiological Science and Technology, 370-1292, Takasaki, Japan
5. Department of Biological Physics, Eötvös University, Pázmány Péter sétány 1/A, H-1117 Budapest, Hungary
6. Wigner Research Centre for Physics, P.O. Box 49, H-1525 Budapest, Hungary
7. Department of Atomic Physics, Budapest University of Technology and Economics, Budafoki út 8., H-1111 Budapest, Hungary

Supplementary Note 1: Saturation behaviour of single h-V_{Si} centre under pulsed laser excitation

To evaluate the optical excitation efficiency in the pulsed regime, we use a 780 nm picosecond laser diode (PicoQuant LDH-P-C-780). The laser operation regime was kept constant to maintain the same pulse shape and duration throughout all measurements. The pulse energy E was subsequently varied and we observed the resulting photon count rate I_{pulsed} in the zero-phonon line, as shown in Supplementary Fig. 1.

In this experiment, we used a slow repetition rate of 2 MHz to assure that the electron comes back to the ground state before laser excitation (and is not trapped in the metastable state with ≈ 100 ns lifetime). Under this condition, we can interpret the background subtracted relative intensity as excitation probability. Since the laser pulse length (56 ps FWHM) is much shorter than the excited state lifetime of h-V_{Si} centre (6 ns), the intensity saturation can be modelled by an exponential equation

$$I_{\mathrm{pulsed}}(E) = I_{0,\mathrm{pulsed}}\left[1 - \exp\left(-\frac{E}{E_0}\right)\right], \quad (1)$$

where $I_{0,\mathrm{pulsed}}$ is the saturation intensity and E_0 is the pulse energy at which the excitation probability is $1 - e^{-1} = 0.632$. We obtained $E_0 = 4.0 \pm 0.1$ pJ from the fitting. The typical laser pulse energy for HOM experiments is 5.5 pJ, which corresponds to an excitation probability of about 74 %.

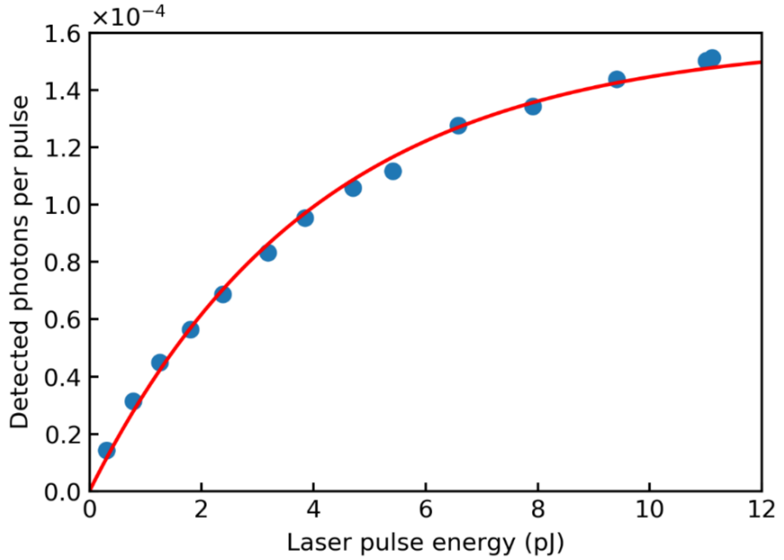

Supplementary Figure 1. Saturation behaviour of photoluminescence intensity in the zero-phonon line pulsed laser excitation at 780 nm. The pulse length is 56 ps in FWHM. The dots are experimental results and the red lines are the fitting to the model Supplementary Eq. (1), yielding a saturation pulse energy of $E_0 = (4.0 \pm 0.1)$ pJ.

Supplementary Note 2: Evaluation of the interferometer — transmissivity/reflectivity ratio of two beam splitters and fringe contrast

In this study, an unbalanced Mach-Zehnder type fibre-based interferometer (Supplementary Fig. 2) is used for HOM type two-photon interference. To characterise the quality of the interferometer, the intensity transmissivity/reflectivity ratio (T/R) of the two beam splitters (BS_1 and BS_2) has to be measured. To this end, we excite a single h-V_{Si} centre by the pulsed laser at 4.1 MHz repetition rate. We detect the rate of ZPL photons through the interferometer by two SNSPDs (D_1 and D_2) in early ($0 \leq t \leq 48$ ns) and late ($\delta t \leq t \leq \delta t + 48$ ns) time bins. Here, $\delta t = 48.7$ ns is the path travel time difference of two arms. As δt is much longer than the photon coherence time, there is no single-photon interference at the output. The photons in the early time bin take the shorter arm and the photons in the late time bin take the longer arm. The integrated photon counts of two detectors in early and late time bins are $N(\mathrm{D}_1, \mathrm{early}) \equiv N_{11} = \eta_1 T_1 R_2 N_0$, $N(\mathrm{D}_1, \mathrm{late}) \equiv N_{12} = \eta_1 R_1 T_2 N_0$, $N(\mathrm{D}_2, \mathrm{early}) \equiv N_{21} = \eta_2 T_1 T_2 N_0$, and $N(\mathrm{D}_2, \mathrm{late}) \equiv N_{22} = \eta_2 R_1 R_2 N_0$, where η_i is the detection efficiency of D_i and N_0 is the total number of input photons. From these relationships, the T/R ratios of two beam splitters are calculated to be $T_1/R_1 = \sqrt{N_{11}N_{21}/N_{12}N_{22}} = 1.129 \pm 0.006$ and $T_2/R_2 = \sqrt{N_{12}N_{21}/N_{11}N_{22}} = 1.046 \pm 0.005$.

The fringe contrast of the interferometer is measured with a highly coherent monochromatic laser (Toptica DLC DL pro 850) at ZPL wavelength of h-V_{Si} centre. By optimising the polarisation rotation in the long interferometer arm with a fibre polarisation controller, the maximum interference fringe contrast obtained in this interferometer is $(1 - \varepsilon) = 0.995$. Since the theoretical limit of the fringe contrast with unbalanced T/R ratios is $2\left(\sqrt{T_1T_2/R_1R_2} + \sqrt{R_1R_2/T_1T_2}\right)^{-1} = 0.9965 \pm 0.0006$, we consider the interferometer to be well aligned.

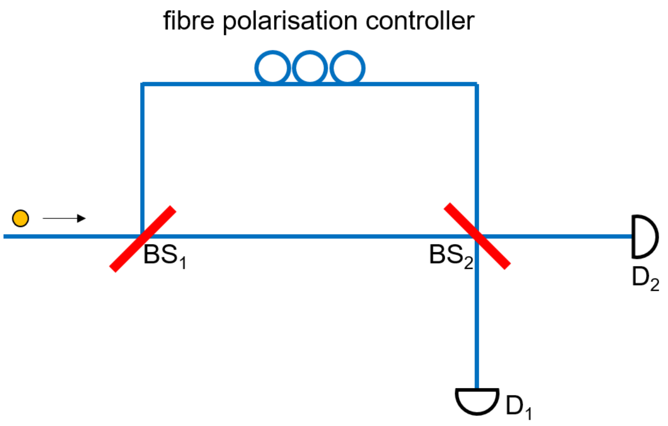

Supplementary Figure 2. Schematic image of the interferometer. $BS_{1,2}$: 50:50 beam splitter, $D_{1,2}$: superconducting nanowire single photon detectors (SNSPD).

Supplementary Note 3: Correction of experimental imperfection factors in Hong-Ou-Mandel interference visibility

The HOM visibility gives the overlap integral of the wave packet of two photons in ideal conditions, but the experiment is affected by timing jitter of photon arrival time, background noise photons, and interferometer imperfections such as non-unity fringe contrast and unbalanced transmissivity and reflectivity. The timing jitter decreases the two-photon overlap integral. The existence of background noise photon decreases the probability of events to have two indistinguishable photons from the h-V_{Si} centre at the beam splitter, resulting in the decrease of a raw HOM visibility. In this note, we extend the discussion on the HOM visibility by Santori et al.[1] by considering the effect of noise photons to estimate the correct photon overlap integral.

The signal photons are from A_1 and A_2 transitions of the h-V_{Si} at the focus and the noise photons come from the ensemble of silicon vacancies on surface, bulk fluorescence, Raman scattering, laser breakthrough, etc.. We denote the probability to have a photon from the signal and noise sources per one laser pulse to be p and q, respectively. Since the pulse length of the excitation laser (56 ps) is much shorter than the excited state lifetime of h-V_{Si} (6 ns), the probability to have two ZPL photons from the same h-V_{Si} centre by one excitation laser is negligibly small. The noise photons can be modelled as a Poissonian photon source, however we can safely neglect the probability to have two noise photons per laser pulse since the average number of noise photon is much smaller than 1 per laser pulse. Under these assumptions, we write the probability to have n photons per laser pulse, p_n $(n = 0, 1, 2)$, and the signal to noise ratio SN as

$$\begin{cases} p_0 = (1-p)(1-q), \\ p_1 = p(1-q) + (1-p)q, \\ p_2 = pq, \end{cases} \tag{2}$$

$$\mathrm{SN} = \frac{p}{q}. \tag{3}$$

Using these parameters and the other mentioned non-ideal parameters, the coincidence counts of five peaks in two-pulse HOM excitation scheme are calculated to be

$$A_0 = (p_1 + 2p_2)^2 \eta_1 \eta_2 N_0 \left\{ T_1 R_1 \left[(T_2^2 + R_2^2) - 2\left(\frac{\mathrm{SN}}{\mathrm{SN}+1}\right)^2 (1-\varepsilon)^2 T_2 R_2 V \right] + 2g(T_1^2 + R_1^2) T_2 R_2 \right\}, \tag{4}$$

$$\begin{cases} A_{+1\cdot\Delta t} = (p_1 + 2p_2)^2 \eta_1 \eta_2 N_0 [(T_1^2 + R_1^2) T_2 R_2 + 2g T_1 R_1 T_2^2], \\ A_{-1\cdot\Delta t} = (p_1 + 2p_2)^2 \eta_1 \eta_2 N_0 [(T_1^2 + R_1^2) T_2 R_2 + 2g T_1 R_1 R_2^2], \end{cases} \tag{5}$$

$$\begin{cases} A_{+2\cdot\Delta t} = (p_1 + 2p_2)^2 \eta_1 \eta_2 N_0 \cdot T_1 R_1 T_2^2, \\ A_{-2\cdot\Delta t} = (p_1 + 2p_2)^2 \eta_1 \eta_2 N_0 \cdot T_1 R_1 R_2^2, \end{cases} \tag{6}$$

where N_0 is the number of repetitions of the experiment, $(1-\varepsilon)$ is the interferometer's fringe contrast, and V is the overlap integral of two photons from the h-V_{Si} centre. The parameter $g = 2p_2/(p_1 + 2p_2)^2$ comes from the events in which two photons enter in the interferometer during one laser excitation pulse. Thus, it is equal to $g^{(2)}(\tau = 0)$ when the autocorrelation measurement is performed under the same condition as the HOM experiment. When $g^{(2)}(\tau = 0)$ degrades solely due to reduced signal-to-noise (SN), this parameter can be written as

$$g = \frac{2pq}{(p+q)^2} = \frac{2\mathrm{SN}}{(\mathrm{SN}+1)^2}. \tag{7}$$

This equation gives the lower limit of the parameter g and $g^{(2)}(\tau = 0)$. Supplementary Fig. 3 shows the comparison of experimentally measured $g^{(2)}(\tau = 0)$ (the same data as Fig. 1(d) in main text), and the SN limited value calculated from Supplementary Eq. (7). At pulse energies below 6 pJ, $g^{(2)}(\tau = 0)$ is close to the SN limit. The degradation of $g^{(2)}(\tau = 0)$ at higher laser pulse energies is probably due to double excitation within one laser pulse. Note that in the HOM experiment, the effect of double excitation is greatly minimised by time-gating in the first several ns.

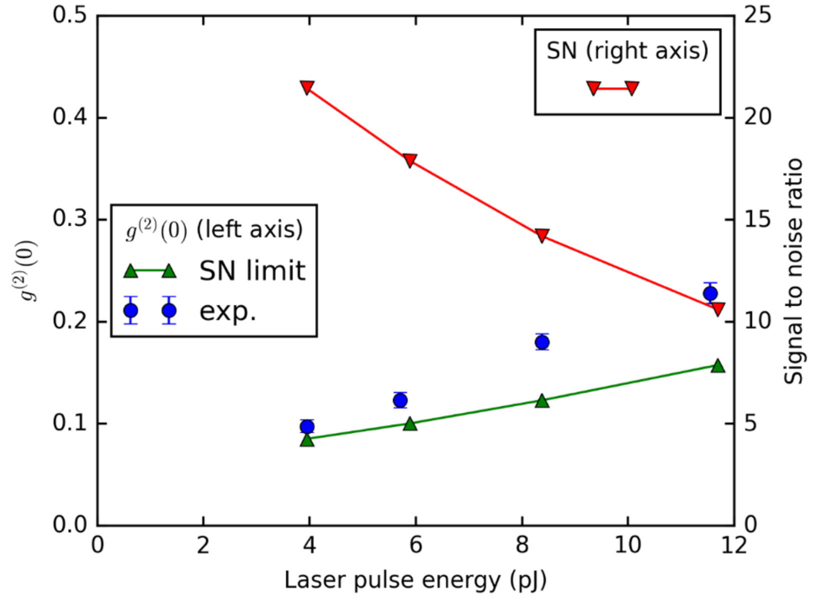

Supplementary Figure 3. $g^{(2)}(0)$ measured with pulsed excitation at repetition rate of 20.5 MHz (blue circles, left axis) and the lower bound of $g^{(2)}(0)$ (green triangles, left axis) calculated using Supplementary Eq. (7) from separately measured signal-to-noise ratio (red inverted triangle, right axis). Lines are guides to the eye. The difference between the experimentally measured $g^{(2)}(0)$ and SN limited one corresponds to $g^{(2)}(0)$ with background correction, i.e., the nonideality of the emitter. Error bars represent one standard error.

From the experimentally obtained raw HOM visibility $V_0 = 1 - 2A_0/(A_{+1\cdot\delta t} + A_{-1\cdot\delta t})$, the corrected HOM visibility (two-photon overlap integral) is extracted to be

$$V = \frac{1}{(1-\varepsilon)^2}\left[\left\{\left(\frac{\mathrm{SN}+1}{\mathrm{SN}}\right)^2 \alpha_2 + \frac{4\alpha_1}{\mathrm{SN}}\right\} - (1 - V_0)\left\{\left(\frac{\mathrm{SN}+1}{\mathrm{SN}}\right)^2 \alpha_1 + \frac{2\alpha_2}{\mathrm{SN}}\right\}\right], \quad (8)$$

$$\alpha_i = \frac{1}{2}\left(\frac{T_i}{R_i} + \frac{R_i}{T_i}\right) \quad (i = 1, 2). \quad (9)$$

Supplementary Fig. 4 shows the reduction of maximally observable experimental visibility due to finite SN ratio. Here, we assume an ideal interferometer ($\varepsilon = 0, \alpha_i = 1$) and perfect two-photon overlap integral ($V = 1$). When SN is 28, which is a typical value for this study at the laser pulse energy of 5.5 pJ, the maximum achievable HOM visibility is upper bound at 81%. Note that for HOM experiments the repetition rate is lower compared to $g^{(2)}(0)$ measurements shown in Supplementary Fig. 3.

Note that SN is a function of the time gating strategy since the noise is composed of photons from various sources with different time scales. For example, the breakthrough of the excitation laser and the Raman scattering are very fast components, but the fluorescence from ensemble of silicon vacancies on surface has the same time constant as the signal photons.

In addition to the visibility reduction due to finite SN, we now consider the effect of timing jitter of the photon arrival time. When the arrival timing of two photons at the second beam splitter is different by $\delta t_{\mathrm{arrival}}$, the visibility decreases by a factor of $\exp(-|\delta t_{\mathrm{arrival}}|/\tau_{\mathrm{ES}})$.[2] By convoluting with a Gaussian distribution of timing jitter ($\text{standard deviation} = \sigma_{\mathrm{jitter}}$), the averaged photon overlap integral decreases by a factor of

$$\beta_{\mathrm{jitter}} = \exp\left[\left(\frac{\sigma_{\mathrm{jitter}}}{\sqrt{2}\tau_{\mathrm{ES}}}\right)^2\right]\mathrm{erfc}\left(\frac{\sigma_{\mathrm{jitter}}}{\sqrt{2}\tau_{\mathrm{ES}}}\right). \quad (10)$$

We estimate the timing jitter of the excitation laser to be about 55 ps (one standard deviation), most of which comes from the trigger pulse generation electronics. By adding the jitter coming from the finite pulse length (56 ps in FWHM), the laser related timing jitter is estimated to be 60 ps (one standard deviation). We assume that the timing jitter caused through the ultra-fast relaxation process in the excited state vibronic levels is negligibly small, thus we take $\sigma_{\mathrm{jitter}} = 60$ ps. As a consequence, the photon overlap integral decreases by 0.8 %. As a result, the HOM visibility after correction (including imperfection of the interferometer, SN ratio, and timing jitter) is

$$V = \frac{1}{2(1-\varepsilon)^2\beta_{\mathrm{jitter}}}\left[\left\{\left(\frac{\mathrm{SN}+1}{\mathrm{SN}}\right)^2 \alpha_2 + \frac{4\alpha_1}{\mathrm{SN}}\right\} - (1 - V_0)\left\{\left(\frac{\mathrm{SN}+1}{\mathrm{SN}}\right)^2 \alpha_1 + \frac{2\alpha_2}{\mathrm{SN}}\right\}\right]. \quad (11)$$

Comparing our theoretical model with the time gated raw data shown in Fig. 4(b) in the main text, we find that the maximum achievable HOM visibility is upper bound at $(80 \pm 1)\%$ by SN, interferometer imperfections and timing jitter. The experimentally extracted visibility parameter $V = 0.85 \pm 0.04$ underlines that essentially ideal contrast could be reached by further improving the setup and noise filtering strategy.

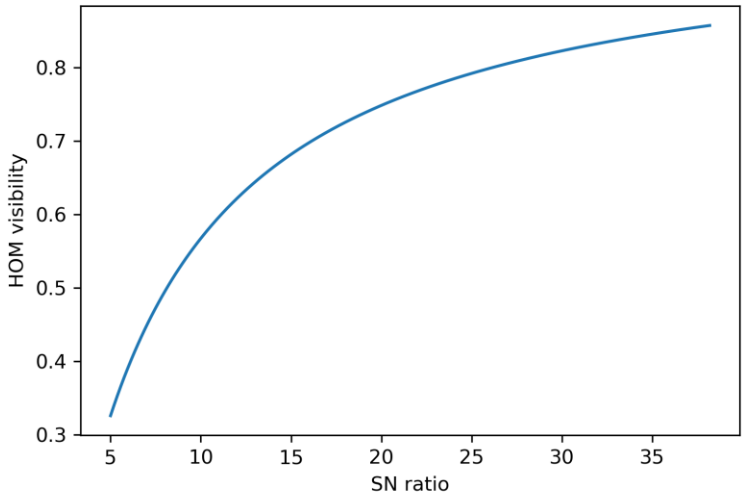

Supplementary Figure 4. Theoretical limit of raw HOM interference visibility in the two-pulse excitation scheme by taking into account the background noise (SN: signal to noise ratio) calculated with Supplementary Eq. (8). The HOM visibility is defined by $1 - 2A_0/(A_{+1\cdot\delta t} + A_{-1\cdot\delta t})$ where A_0 is the coincidence at zero time delay and $A_{\pm 1\cdot\delta t}$ are those at the time delay of $\pm\delta t$. In this plot, unity two-photon overlap integral and ideal interferometer are assumed.

Supplementary Note 4: Resonant Rabi oscillation experiment with short radiofrequency pulse for spin-controlled indistinguishable photon generation

To control the colour of photons via coherent manipulation of the ground state spin, a radiofrequency (RF) pulse is applied to the centre between two laser excitation pulses. The first optical transition initialises the spin state into one of the Kramers doublet subspaces $m_s = \pm 1/2$ or $\pm 3/2$ depending on the observed colour of the emitted zero phonon line photon (A_1 or A_2, respectively). The RF pulse coherently manipulates the spin state, and the resulting spin population in each subspace directly translated to the probability to observe the second photon in A_1 or A_2. Considering the time difference of interferometer arms $\delta t = 48.7$ ns and the system's excited state lifetime $\tau_{ES} = 6$ ns, the allowed maximum pulse length is about 30 ns. Due to the short pulse length, we expect a frequency broadening exceeding the ground state zero-field splitting (ZFS) of 4.5 MHz. Thus, the RF field will drive all spin transitions simultaneously, leading to spin manipulation with non-unity fidelity. To determine optimal RF pulse length and the associated spin populations, we measured Rabi oscillation with resonant laser excitation[3].

The h-V_{Si} centre is irradiated by a laser resonant to the A_2 (A_1) transition for 9 μs, which initialises the spin state into $m_s = \pm 1/2$ ($\pm 3/2$) subspace. Thereafter, a RF pulse resonant with the $|3/2\rangle_{GS} \leftrightarrow |1/2\rangle_{GS}$ transition is applied. Subsequently, the population of the spin sublevels $m_s = \pm 3/2$ ($\pm 1/2$) is read out by the same laser via the fluorescence intensity in the first 500 ns. Supplementary Figs. 5 (a) and (b) show the normalised results of Rabi experiment at RF power of 30 dBm. Due to the high pulse power, the Rabi frequency exceeds the zero-field splitting, resulting in an oscillation pattern that is different from a simple cosine function. However, we still observe oscillations due to spin population flips (a detailed theoretical model of this Rabi result will be given later in this Supplementary Note). We determine three relevant pulse durations: 10 ns to induce a $\pi/4$-pulse, 19 ns to induce a $\pi/2$-pulse, and 29 ns to induce a $3\pi/4$-pulse.

Due to the high-power RF condition the sample heats up significantly, which causes optical line broadening. Supplementary Fig. 5(c) shows typical optical absorption spectra (photoluminescence excitation spectroscopy) measured under similar RF conditions as used during the HOM experiments. To this end, we apply RF pulses with 30 dBm power at a repetition cycle of $10 \cdot \delta t = 487$ ns. For RF pulse durations of 10 ns, 19 ns, and 29 ns, we observe linewidths of 82 ± 1 MHz, 86 ± 1 MHz, 112 ± 2 MHz. Although not implemented here, we note that this heating can be minimised by optimising the structure of the RF antenna to obtain a better RF field coupling to the spin.

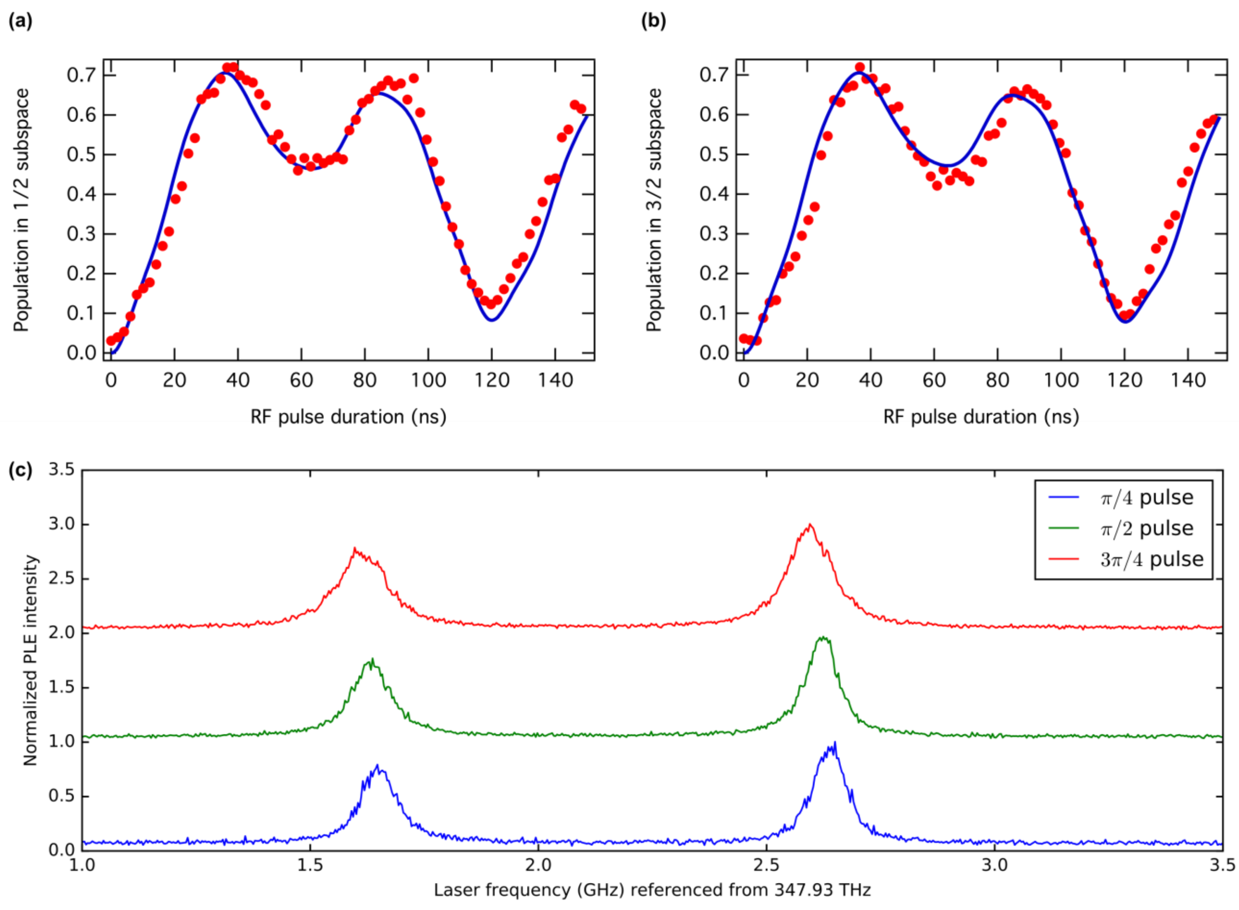

Supplementary Figure 5. Typical experimental data for Rabi oscillations under strong RF driving field, resonant with the $|3/2\rangle_{\mathrm{GS}} \leftrightarrow |1/2\rangle_{\mathrm{GS}}$ transition. (a) Spin population development after the system is initialised into the $m_s = \pm 1/2$ subspace. (b) Spin population development after the system is initialised into the $m_s = \pm 3/2$ subspace. Red dots are data, and blue lines are fits. (c) Optical absorption spectra measured by photoluminescence excitation spectroscopy under the existence of RF pulses at the repetition cycle of 487 ns. The laser power was set to $3.5\ \mathrm{W} \cdot \mathrm{cm}^{-2}$ to reduce laser power broadening. For the $\pi/4, \pi/2$ and $3\pi/4$ pulses, the observed linewidths are 82 ± 1 MHz, 86 ± 1 MHz, 112 ± 2 MHz, respectively (average over both transitions).

For proper interpretation of the visibility of the spin-controlled HOM interference experiments, we quantify the maximally achievable HOM visibility at those different RF pulse and temperature conditions. To this end, we always perform an additional HOM interference experiment in which the identical RF pulse is applied right before an experimental sequence, instead of applying it during the sequence. A typical measurement with uncorrected data is shown in Supplementary Fig. 6. Then we obtain the normalised HOM visibility as

$$V_{\text{norm}} = \frac{V(\text{RF during sequence})}{V(\text{RF before squence})}. \tag{12}$$

Here, $V(\text{RF during sequence})$ and $V(\text{RF before squence})$ are corrected for experimental imperfections by Supplementary Eq. (11). This way, the value $(1 - V_{\text{norm}})$ equals to the amount of spin population flip from the initial spin subspace to the other, which can be directly compared with Rabi oscillation result as shown in Fig. 3(d) in the main text.

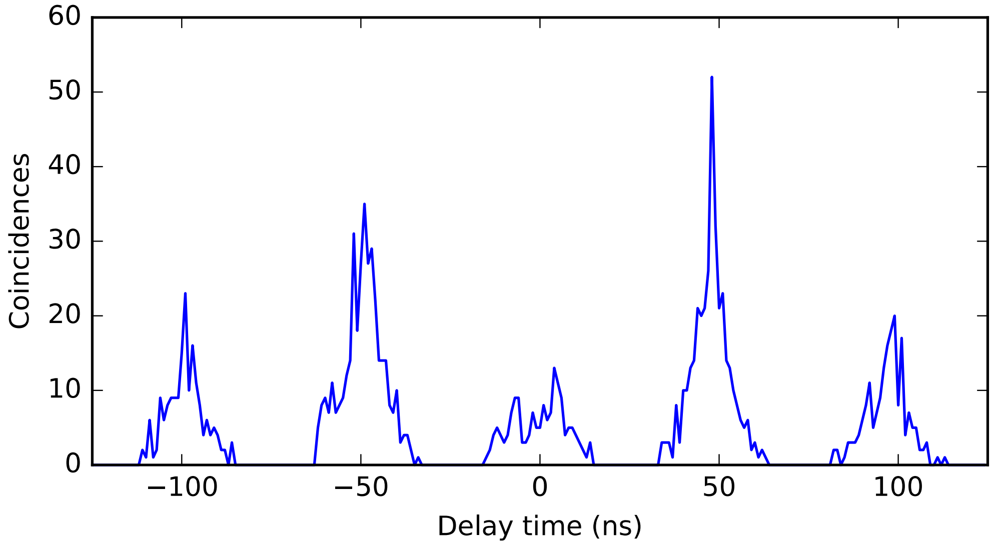

Supplementary Figure 6. Reference HOM measurement with $\frac{\pi}{2}$ pulse before the experimental sequence. Time gating settings are $t_{\text{Start}} = 2$ ns and $\Delta t = 16$ ns. The observed raw HOM visibility is 0.56 ± 0.04. After experimental imperfection corrections, we obtain $V(\text{RF before sequence}) = 0.73 \pm 0.05$.

Time evolution of ground state spin populations under pulsed strong RF drive

To evaluate the time-dependent spin populations in the ground states under strong RF drive, we start with the static Hamiltonian, describing the system in an external magnetic field aligned along the z-axis:

$$H_0 = D \cdot S_z^2 + \gamma_e \cdot B_z \cdot S_z. \tag{13}$$

Here, $D = 2\pi \cdot 2.25\,\text{MHz}$ is the ground state zero-field splitting, $\gamma_e = 2\pi \cdot 28\,\text{GHz} \cdot \text{T}^{-1}$ the electron gyromagnetic ratio, and $B_z \approx 0.9$ mT the externally applied field. Our RF drive is modelled by the interaction Hamiltonian:

$$H_{\text{RF}} = \Omega \cdot \cos(2\pi f t + \phi) \cdot S_x \tag{14}$$

Here, $\Omega \approx 2\pi \cdot 14$ MHz is the strength of the RF driving field, $f = 30.26911\text{MHz}$ is the RF frequency, t the time, and ϕ is a (random) phase term, which accounts for the fact that the RF driving is here faster than the ground state level separation $(2 \cdot D)$ and not phase-synchronised, thus the rotating wave approximation might not be valid. With these Hamiltonian operators, the time evolution operator is given by

$$U(t) = e^{-i\int_0^t [H_0 + H_{\text{RF}}(t')]dt'}. \tag{15}$$

The time evolution of the four spin states in the ground state is then

$$\rho(t) = U(t)\rho_0 U^\dagger(t). \tag{16}$$

Here, $\rho(t)$ is the density matrix describing the system at time t and ρ_0 is the state at $t = 0$. We obtain $\rho(t)$ by solving Supplementary Eq. (15) numerically, averaging over the random phase term ϕ, and using small time steps $dt \approx 0.5$ ns, which is significantly smaller than the typical time scale of spin state development (≈ 10 ns).

For the spin-controlled HOM interference experiments, emission of the first photon in the A_1 or A_2 line projects the state into the $m_s = \pm\frac{1}{2}$ or $m_s = \pm\frac{3}{2}$ ground state spin subspace, respectively. Thus, we have to distinguish between two realisations in which $\rho_0 = \frac{1}{2}\left(\left|\frac{1}{2}\right\rangle\left\langle\frac{1}{2}\right| + \left|-\frac{1}{2}\right\rangle\left\langle-\frac{1}{2}\right|\right) \equiv \rho_0^{(1/2)}$ or $\rho_0 = \frac{1}{2}\left(\left|\frac{3}{2}\right\rangle\left\langle\frac{3}{2}\right| + \left|-\frac{3}{2}\right\rangle\left\langle-\frac{3}{2}\right|\right) \equiv \rho_0^{(3/2)}$, respectively. Supplementary Figs. 5 (a) and (b) shows the comparison of our theoretical model with the experimental results of resonant Rabi oscillation (described in Supplementary Note 4) with the initial state of $\rho_0^{(3/2)}$ and $\rho_0^{(1/2)}$, respectively. To fit the data to our model, we normalise the amplitude of both the experimental data and theoretical model. We solve Supplementary Eq. (16) with two free parameters, i.e. Ω and B_z, and minimise error squares. From both data sets, we extract a Rabi frequency of $\Omega = 2\pi \cdot (14.4 \pm 0.2)$ MHz and $B_z = 0.919 \pm 0.003$ mT. In the end, we correct both the experimental data and the theoretical model for the above-used amplitude normalisation factor.

In the HOM experiment under off-resonant excitation, each individual initial spin-state subspace is randomly chosen with almost equal probability after the system experiences intersystem crossing[3]. The resulting HOM visibility is an average of the cases with two different initial states. However, the data and fits show that the spin population transfer from each spin subspace to the other is almost equally efficient. Therefore, we can directly model the normalised HOM visibility as $V_{\text{norm}} = 1 - \bar{p}$, in which $\bar{p}$ is the amount of flipped spin population,

after averaging over both realisations (i.e. with the system being in the initial state of $\rho_0^{(3/2)}$ and $\rho_0^{(1/2)}$, respectively). The associated model is plotted as a solid line in Fig. 3(d) in the main text.

Supplementary Note 5: Analysis of pure dephasing rate and spectral diffusion amplitude by HOM visibility with time-gating

First, we derive the Eq. (1) in the main text. We follow the derivation given by Thoma et al.[4]. Assume the photons arrive at the beam splitter at $t = 0$ and detected at $t = t_{\mathrm{D}}$ and $t = t_{\mathrm{D}} + \tau$ at different detectors. The time-resolved coincidence count rate per a pair of photons is given by

$$G^{(2)}(t_D, \tau) = \Gamma^2 (1 - e^{-\gamma\tau}) \mathrm{e}^{-\Gamma(2t_{\mathrm{D}}+\tau)}. \tag{17}$$

Here, $\Gamma = \frac{1}{6\ \mathrm{ns}}$ is the inverse excited state lifetime, and $\gamma = \Gamma_0' \left[1 - \mathrm{e}^{-(\delta t/\tau_{\mathrm{c}})^2}\right] + 2\gamma'$, with Γ_0' being the amplitude of spectral diffusion, τ_{c} the associated time constant, and γ' the pure dephasing rate of the single emitter[5]. Also, we consider ideal 50:50 beam splitter since we compare this theory with experimental HOM visibility after imperfection correction by Supplementary Eq. (11). Integration of Supplementary Eq. (17) over t_{D} and τ within the gated detection time window $\left[t_{\mathrm{Start}}, t_{\mathrm{Stop}}\right]$ gives the HOM visibility after normalisation. The coincidence for normalisation is given by

$$G'^{(2)}(t_{\mathrm{D}}, \tau) = \Gamma^2 \mathrm{e}^{-\Gamma(2t_{\mathrm{D}}+\tau)}. \tag{18}$$

Therefore, the HOM visibility with time gating analysis is calculated to be

$$V = 1 - \frac{\int_{t_{\mathrm{Start}}}^{t_{\mathrm{Stop}}} dt_{\mathrm{D}} \int_0^{t_{\mathrm{Stop}}-t_{\mathrm{D}}} d\tau G^{(2)}(t_{\mathrm{D}}, \tau)}{\int_{t_{\mathrm{Start}}}^{t_{\mathrm{Stop}}} dt_{\mathrm{D}} \int_0^{t_{\mathrm{Stop}}-t_{\mathrm{D}}} d\tau G'^{(2)}(t_{\mathrm{D}}, \tau)}, \tag{19}$$

which equals to Eq. (1) in the main text.

By fitting the data in main text Fig. 4(c) with the model in Eq. (1), we extract γ (which is the only free fitting parameter). For further analysis, we decompose γ into a slow term (related to spectral diffusion with amplitude Γ_0'), and a fast term (related to pure dephasing with rate γ'). As outlined in the main text, the major contribution to HOM visibility reduction is pure dephasing, while spectral diffusion due to laser ionisation is relatively slow compared to the experimental time scale, i.e. $\tau_c \gg \delta t = 48$ ns. Then we can directly extract the maximum pure dephasing rate, as it is $\gamma = 2\gamma'_{\max}$. In other words, HOM visibility reduction is only due to pure dephasing. The amplitude of (slow) spectral diffusion Γ_0' is obtained by considering the emission linewidth (measured over time scales of seconds to minutes). The FWHM optical linewidth is

$$\Delta\nu = \frac{\Gamma + \Gamma_0' + \gamma'}{2\pi}. \tag{20}$$

For reasons of completeness, we can also assume the opposite scenario, i.e. no pure dephasing ($\gamma' = 0$ MHz), such that HOM visibility contrast reduction is solely explained by fast spectral diffusion. In this case we compute the maximum spectral diffusion amplitude $\Gamma'_{0,\mathrm{max}}$ according to Supplementary Eq. (20), and solve $\gamma = \Gamma_0'\left[1 - \mathrm{e}^{-(\delta t/\tau_{\mathrm{c,min}})^2}\right]$ for the minimal spectral diffusion time constant $\tau_{\mathrm{c,min}}$.

The related results are shown in the in Supplementary Table I.

Supplementary Table I. Summary of temperature dependent spectral linewidth (averaged over both transitions A_1 and A_2), the maximum pure dephasing rate γ'_{max} and associated spectral diffusion amplitude Γ_0' for the model in which the HOM contrast is limited by pure dephasing. Additionally, we give the maximum amplitude of spectral diffusion $\Gamma'_{0,\mathrm{max}}$ and its minimum time constant $\tau_{\mathrm{c,min}}$ for the model in which the HOM contrast is solely limited by spectral diffusion (i.e. $\gamma' = 0$). Error values correspond to one standard error.

		Pure dephasing limited		Spectral diffusion limited	
Temperature [K]	PLE linewidth [MHz]	$\gamma'_{\mathrm{max}}/2\pi$ [MHz]	$\Gamma_0'/2\pi$ [MHz]	$\Gamma'_{0,\mathrm{max}}/2\pi$ [MHz]	$\tau_{\mathrm{c,min}}$ [ns]
5.0	62.4 ± 0.4	1.5 ± 0.4	34.4 ± 0.5	35.9 ± 0.4	109 ± 8
5.9	70.1 ± 0.3	4.0 ± 0.7	39.6 ± 0.7	43.6 ± 0.3	81 ± 6
6.8	82.4 ± 0.3	11.5 ± 1.7	44.4 ± 1.8	55.9 ± 0.3	51 ± 6

Supplementary Note 6: Vibronic interaction theory

The origin of dephasing in the optical signal for V1 center is the coupling to the V1' polaronic excited state mediated by acoustic phonons. As outlined in more details in Udverhelyi et al.[6], at very low temperatures, only the acoustic phonons have significant occupation number. However, compared to the temperatures in the experiment, the polaronic gap between V1 and V1' is relatively large (4.4 meV), which excludes the consideration of two-phonon (Raman scattering) processes to be competitive with the single phonon absorption process. Thus we describe the dephasing with a resonant phonon coupling[7]. This can be formulated using time-dependent perturbation theory with first order contribution in the linear vibronic interaction leading to Fermi's Golden Rule formula for the transition rate

$$\gamma' = 2\pi \sum_k n_k \, |\chi_k|^2 \delta(\Delta E - \hbar\omega_k), \tag{21}$$

where k is the index of phonon mode, n_k is the acoustic phonon occupation number, χ_k is the linear vibronic interaction strength, $\hbar\omega_k$ is the acoustic phonon energy, and ΔE is the energy gap between V1 and V1' levels. For the density of acoustic phonon states $\rho(\omega)$ we use the Debye-model as $\rho(\omega) = \rho\omega^2$, where ρ is a constant. We can approximate $\overline{|\chi_k|^2} \approx \chi\omega$ phonon mode average for the acoustic phonons, where χ is a constant. After this, the summation results in

$$\gamma' = \frac{2\pi}{\hbar^3} \rho\chi(\Delta E)^3 n(\Delta E, T), \tag{22}$$

where we use the thermal occupation function of phonons $n(\Delta E, T)$. Since ΔE is relatively large we find the low temperature limit of this function with exponential temperature dependence, as described in Eq. (2) in the main text.

Supplementary Note 7: Analysis of quantum beating with spin control via RF pulse

This note explains the analysis of quantum beating obtained with the HOM interference experiment with spin-flip RF pulses. The time delay of the RF pulse from the first laser pulse is 18 ns. Therefore, the coincidence data is taken within the detection time window $[t_{\mathrm{Start}}, t_{\mathrm{Stop}}] = [1.5\ \mathrm{ns}, 18\ \mathrm{ns}]$ with the time-gating technique described in Fig. 4(a) in the main text. This strategy rejects the laser related noise and ensures that the system is in the ground state while RF pulse is applied for the collected data. Due to non-unity spin flip fidelity in our conditions and the existence of noise photons, we consider three components in the coincidence data. The first component is the interference of photons from different transitions $\{A_1, A_2\}$, which causes beating due to the frequency difference of two photons $\delta\nu \cong 1$ GHz. The coincidence count distribution per pair of two photons (detected at $t = t_{\mathrm{D}}$ and $t = t_{\mathrm{D}} + \tau$ at different detectors, $t = 0$: the earliest possible arrival time) for this case is given by[4,5]

$$G_1^{(2)}(t_D, \tau) = \Gamma^2[1 + \cos(2\pi\delta\nu\tau + \pi)\, \mathrm{e}^{-\gamma\tau}]\mathrm{e}^{-\Gamma(2t_{\mathrm{D}}+\tau)}, \tag{23}$$

where $\Gamma = \frac{1}{6\ \mathrm{ns}}$ is the inverse excited state lifetime, γ is the sum of the pure dephasing rate and the spectral diffusion rate discussed in Supplementary Note 5. The integration of the Supplementary Eq. (23) within the detection time window $t_{\mathrm{D}} \in [t_{\mathrm{Start}}, t_{\mathrm{Stop}} - \tau]$ gives the beating pattern in the correlation measurement

$$\bar{G}_1^{(2)}(0 < \tau < \Delta t) = \int_{t_{\mathrm{Start}}}^{t_{\mathrm{Stop}}-\tau} dt_{\mathrm{D}} G_1^{(2)}(t_{\mathrm{D}}, \tau)$$

$$= \frac{\Gamma}{2}\mathrm{e}^{-2\Gamma t_{\mathrm{Start}}} e^{-\Gamma\tau}\left[1 - \mathrm{e}^{-2\Gamma(\Delta t - \tau)}\right][1 + \cos(2\pi\delta\nu\tau - \pi)\, \mathrm{e}^{-\gamma\tau}]. \tag{24}$$

Here, $\Delta t = 16.5$ ns is the detection window. The second component is the interference of photons from the same transition $\{A_i, A_i\}$ $(i = 1, 2)$, which results from non-perfect spin flip fidelity by RF pulse. The corresponding correlation data $\bar{G}_2^{(2)}(\tau)$ is obtained by substituting $\delta\nu = 0$ to Supplementary Eq. (24)

$$\bar{G}_2^{(2)}(\tau) = \frac{\Gamma}{2}\mathrm{e}^{-2\Gamma t_{\mathrm{Start}}}\mathrm{e}^{-\Gamma\tau}\left[1 - \mathrm{e}^{-2\Gamma(\Delta t - \tau)}\right][1 - \mathrm{e}^{-\gamma\tau}]. \tag{25}$$

For simplicity, we assume γ in Supplementary Eqs. (24) and (25) are approximated to be the same. The third component is the interference involves noise photons and remained coincidences resulting from the imperfection of the interferometer. Here, we assume that noise photons have the same decay time as the signal photon, which is indeed observed in the experiment and is probably from an h-V_{Si} ensemble that exists on the surface. The fast noise photons (laser related) are filtered out by time-gating. The frequency difference of photons involving noise photons is randomly distributed and the optical coherence time is expected to be much shorter than the timing

resolution of our detection system and electronics (0.4 ns). Therefore, the corresponding coincidence counts $\bar{G}_3^{(2)}$ is obtained from Supplementary Eq. (24) by averaging cosine term and taking a limit of $\gamma \to \infty$ as

$$\bar{G}_3^{(2)}(\tau) = \frac{\Gamma}{2} \mathrm{e}^{-2\Gamma t_{\mathrm{Start}}} \mathrm{e}^{-\Gamma\tau} \left[1 - \mathrm{e}^{-2\Gamma(\Delta t - \tau)}\right]. \tag{26}$$

The total coincidence counts $\bar{G}_{\mathrm{tot}}^{(2)}$ are obtained by summing up these three components $\bar{G}_i^{(2)}$ with associated coefficients c_i. By considering $\tau < 0$ region, a small time difference between two detectors t_0, and the finite detection timing resolution of the detectors and electronics (approximated by an Gaussian broadening with a standard deviation of σ_{det}), the total coincidence is calculated to be

$$\bar{G}_{\mathrm{tot}}^{(2)}(\tau) = \left[\sum_{i=1}^{3} c_i \bar{G}_i^{(2)}(|\tau - t_0|)\right] * \left[\frac{1}{\sqrt{2\pi}\sigma_{\mathrm{det}}} \exp\left(-\frac{\tau^2}{2\sigma_{\mathrm{det}}^2}\right)\right]. \tag{27}$$

This function is fitted to the experimental data which is measured with 0.1 ns bin width and is smoothened by three-point averaging (the time binning and 3 point average are also considered in the fitting). In Supplementary Eq. (23), the non-ideal transmissivity/reflectivity ratio of the beam splitters and the non-unity interferometer fringe contrast is neglected. However, these imperfections are considered in the evaluation of the coefficient c_3.

We can extract the parameters γ, c_2/c_1, and c_3/c_1 from experiments. $\gamma = (84 \pm 4)$ MHz is obtained from the HOM visibility when the RF pulse is applied prior to the first laser excitation pulse (the experiment without spin flip). The coefficient ratio $c_2/(c_1 + c_2)$ corresponds to the amount of the spin population transfer by the RF pulse, which is equal to $(1 - V_{\mathrm{norm}}) = 0.37 \pm 0.06$ as discussed in Supplementary Note 4. As a result, we obtain

$$\frac{c_2}{c_1} = \frac{V_{\mathrm{norm}}}{1 - V_{\mathrm{norm}}} = 1.68 \pm 0.45. \tag{28}$$

The coefficient ratio $c_3/(c_1 + c_2)$ can be evaluated from Supplementary Eq. (4)

$$\frac{c_3}{c_1 + c_2} = \frac{A_0|_{V=1}}{A_0|_{V=0} - A_0|_{V=1}} = \frac{\alpha_2 + 2\alpha_1 g}{[\mathrm{SN}/(\mathrm{SN}+1)]^2 (1-\varepsilon)^2} - 1 \tag{29}$$

as $A_0|_{V=1}$ corresponds to the coincidence count remained when the photons from the emitter is perfectly indistinguishable, i.e., contributions from noise photons and the interferometer imperfections, and the rest is the possible number of events related to the interference of photons from $\{A_i, A_j\}$ transitions. In combination with Supplementary Eq. (29), we obtain $c_3/c_1 = 0.63 \pm 0.11$. With these fixed parameters, there are only four fitting parameters left: $c_1 = 753 \pm 12$, $t_0 = (0.00 \pm 0.02)$ ns, $\delta\nu = (0.965 \pm 0.006)$ GHz, and $\sigma_{\mathrm{det}} = (0.17 \pm$

$0.02)$ ns. The beating frequency $\delta\nu$ well agrees with the frequency difference of A_1 and A_2 transitions and the detection timing resolution σ_{det} is reasonable considering the SNSPD manufacturer's specifications.

Supplementary Note 8: ZPL fine structure investigations with Fabry-Pérot filter cavity

To investigate the ZPL emission linewidth under off-resonant excitation, we use a home-made fibre-coupled narrowband plano-convex Fabry-Pérot cavity as high-resolution spectrometer for the ZPL emission.

In the first step, we characterise the main cavity parameters, i.e. its free spectral range (FSR) and transmission linewidth $\Delta\nu_{\mathrm{cav}}$. To this end, we scan a narrowband tunable diode laser (Toptica DLpro) over several cavity transmission peaks. The frequency of the laser is constantly monitored using a wavemeter (HighFinesse WS7-30). The result is shown in Supplementary Fig. 7.

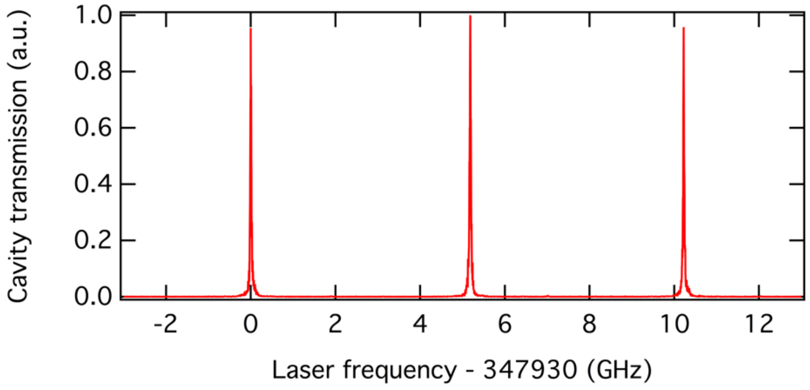

Supplementary Figure 7. Cavity transmission while scanning a narrowband laser over several transmission peaks.

We find that the average separation between two consecutive peaks is $\mathrm{FSR} = 5.145$ GHz, and that the Lorentzian transmission width of each individual peak is $\Delta\nu = 29 \pm 1$ MHz.

Having characterised the main cavity parameters, we now use it as a spectrometer for inferring the linewidth of the ZPL emission in the A_1 and A_2 transitions under off-resonant excitation. We use continuous-wave off-resonant excitation at 730 nm, and at a power of 150 μW, which corresponds to approximately three times the saturation power (half of maximum count rate is reached at $P_{\mathrm{sat}} = 57 \pm 5$ μW). We split the h-V_{Si} centre emission into PSB and ZPL using a dichroic mirror. The ZPL emission is then fibre-coupled and sent to the cavity. We tune the cavity length (and thus the transmission frequency) via a piezoelectric actuator attached to one of the cavity mirrors. To monitor slow cavity drifts (e.g. due to temperature), we use the narrowband resonant excitation laser as a reference. The laser is frequency locked using the wavemeter at a frequency of 1-2 GHz below the h-V_{Si} transition lines. A small fraction of the laser is sent through the setup, and through the cavity filter. A typical measurement result is shown in Supplementary Fig. 8.

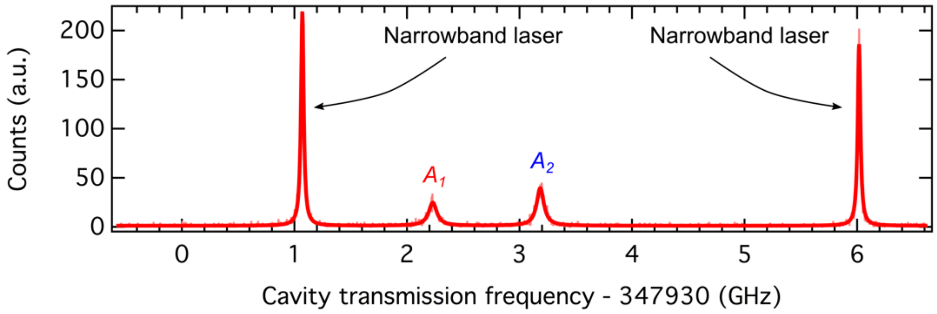

Supplementary Figure 8. Typical cavity-recorded emission spectrum of the single h-V_{Si} centre used for the HOM experiments. Four peaks are observed, and associated with the narrowband frequency reference laser, and the A_1 and A_2 transitions, respectively.

The narrowband cavity is scanned in length for a frequency equivalent of about 7 GHz. The two high peaks correspond to the frequency-stabilised narrowband laser. From the previous cavity characterisation, the peak separation is known to be 5.145 GHz, which provides the necessary frequency calibration for the measurement. The two smaller peaks are associated with the A_1 and A_2 transitions, respectively. Fitting the data with four Lorentzian peaks allows to extract the emission linewidths of the A_1 and A_2 transitions, respectively. Due to the finite resolution of the Fabry-Pérot cavity ($\Delta\nu = 29 \pm 2$ MHz), the emission linewidths of the two transitions appear larger than they are. Thus, we deconvolute the measured linewidth with the cavity linewidth, using the fact that the convolution of two Lorentzian functions with widths a_1 and a_2 results in a Lorentzian function with width $a = a_1 + a_2$. For the defect that was used for the HOM experiments, we infer deconvoluted linewidths of $\Delta\nu_1 = 57 \pm 6$ MHz and $\Delta\nu_2 = 48 \pm 6$ MHz for the A_1 and A_2 transitions, respectively.

To show the repeatability of our results, we perform the same studies on six different h-V_{Si} centres. The emission spectra are shown in Supplementary Fig. 9.

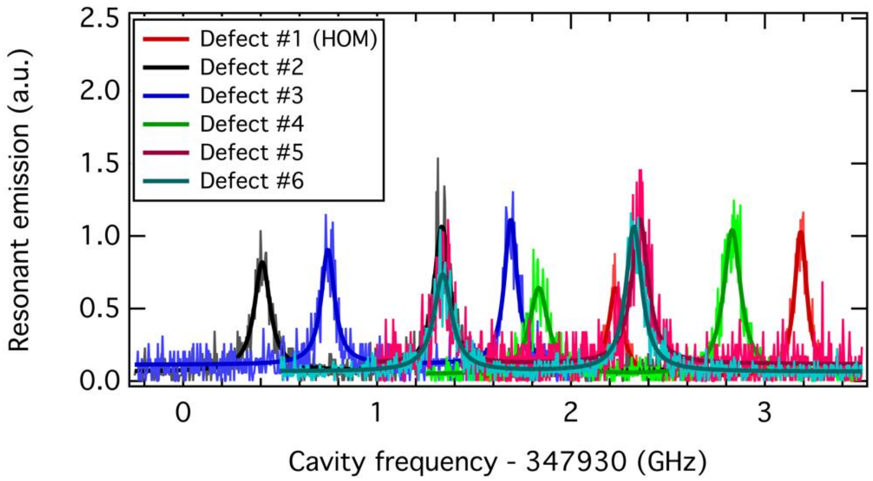

Supplementary Figure 9. Emission spectra of six h-V_{Si} centres. HOM experiments have been performed on defect #1. However, all other observed defects show basically the same narrow emission lines, underlining the repeatability of the experiment. The A_1 transition linewidths were found in the range of $49 - 89$ MHz, while the A_2 emission linewidths are found in the range of $46 - 76$ MHz (after correcting for the finite resolution of the Fabry-Pérot cavity spectrometer). The separation between two lines (mainly due to excited state zero field splitting) is also very consistent and in the range of $0.927 - 1.015$ GHz. All solid lines are fits to the data using Lorentzian functions.

Supplementary References

1. Santori, C., Fattal, D., Vuckovic, J., Solomon, G. S. & Yamamoto, Y. Indistinguishable photons from a single-photon device. *Nature* **419**, 594–597 (2002).

2. Fearn, H. & Loudon, R. Theory of two-photon interference. *J. Opt. Soc. Am. B* **6**, 917–927 (1989).

3. Nagy, R. et al. High-fidelity spin and optical control of single silicon-vacancy centres in silicon carbide. *Nat. Commun.* **10**, 1954 (2019).

4. Thoma, A. et al. Exploring Dephasing of a Solid-State Quantum Emitter via Time- and Temperature-Dependent Hong-Ou-Mandel Experiments. *Phys. Rev. Lett.* **116**, 033601 (2016).

5. Kambs, B. & Becher, C. Limitations on the indistinguishability of photons from remote solid state sources. *New J. Phys.* **20**, 115003 (2018).

6. Udvarhelyi, P. et al. Vibronic states and their effect on the temperature and strain dependence of silicon-vacancy qubits in 4H silicon carbide. *Phys. Rev. Appl.* in press. (2020).

7. Jahnke, K. D. et al. Electron-phonon processes of the silicon-vacancy centre in diamond. *New J. Phys.* **17**, 043011 (2015).
